# Decreased parenchymal arteriolar tone uncouples vessel-to-neuronal communication in a mouse model of vascular cognitive impairment

**DOI:** 10.1007/s11357-020-00305-x

**Published:** 2021-01-07

**Authors:** Ki Jung Kim, Juan Ramiro Diaz, Jessica L. Presa, P. Robinson Muller, Michael W. Brands, Mohammad B. Khan, David C. Hess, Ferdinand Althammer, Javier E. Stern, Jessica A. Filosa

**Affiliations:** 1 Department of Physiology, Augusta University, Augusta, GA 30912 USA; 2 Department of Neurology, Medical College of Georgia, Augusta University, Augusta, GA USA; 3 Neuroscience Institute, Georgia State University, Atlanta, GA USA

**Keywords:** Hypoperfusion, Neurovascular unit, Adenosine, Myogenic tone, A1R

## Abstract

Chronic hypoperfusion is a key contributor to cognitive decline and neurodegenerative conditions, but the cellular mechanisms remain ill-defined. Using a multidisciplinary approach, we sought to elucidate chronic hypoperfusion-evoked functional changes at the neurovascular unit. We used bilateral common carotid artery stenosis (BCAS), a well-established model of vascular cognitive impairment, combined with an ex vivo preparation that allows pressurization of parenchymal arterioles in a brain slice. Our results demonstrate that mild (~ 30%), chronic hypoperfusion significantly altered the functional integrity of the cortical neurovascular unit. Although pial cerebral perfusion recovered over time, parenchymal arterioles progressively lost tone, exhibiting significant reductions by day 28 post-surgery. We provide supportive evidence for reduced adenosine 1 receptor-mediated vasoconstriction as a potential mechanism in the adaptive response underlying the reduced baseline tone in parenchymal arterioles. In addition, we show that in response to the neuromodulator adenosine, the action potential frequency of cortical pyramidal neurons was significantly reduced in all groups. However, a significant decrease in adenosine-induced hyperpolarization was observed in BCAS 14 days. At the microvascular level, constriction-induced inhibition of pyramidal neurons was significantly compromised in BCAS mice. Collectively, these results suggest that BCAS uncouples vessel-to-neuron communication—vasculo-neuronal coupling—a potential early event in cognitive decline.

**Supplementary Information:**

The online version contains supplementary material available at 10.1007/s11357-020-00305-x.

## Introduction

Carotid artery stenosis compromises cerebral blood flow (CBF), giving rise to chronic hypoperfusion and, over time, cognitive decline. Chronic hypoperfusion is observed in multiple central nervous system diseases, including multiple sclerosis [1, 2], Parkinson’s [3] and Alzheimer’s diseases [4], and vascular dementia [5], and is a consequence of aging [6, 7]. It is also associated with risk factors such as atherosclerosis, obesity, and hypertension [8]. Importantly, brain function requires constant CBF, a requirement that depends on optimal signaling at the neurovascular unit (NVU), comprising neurons, astrocytes, microglia, pericytes, endothelial cells, and vascular smooth muscle cells. Thus, understanding the impact of chronic hypoperfusion on the integral function of the NVU is of critical importance [9].

Cells of the NVU work in concert to ensure brain homeostasis [10]. Ischemic conditions can disrupt NVU homeostasis, leading to a number of pathological changes, including increases in blood-brain barrier (BBB) permeability, activation of inflammatory pathways, neuronal excitotoxicity, and astrocyte dysfunction (e.g., impaired K^+^ homeostasis, Ca^2+^ overload). In disease states, chronic hypoperfusion may trigger activation of neuroprotective or adaptive processes that serve to compensate for the initial disturbance (i.e., ischemia). With time, however, these pathways may desensitize or become aberrant, causing the transition to a maladaptive state in which dysfunction and pathological symptoms prevail.

We previously reported that, in parenchymal arterioles, a rise in intravascular pressure increases astrocytic Ca^2+^ and reduces resting cortical pyramidal neuron firing activity at the NVU [11]. We further showed that the reduction in pyramidal neuron firing was primarily attributable to activation of adenosine 1 receptors (A1Rs). These findings led us to propose that, in the healthy brain, coupling of parenchymal arteriole myogenic constriction with a reduction in pyramidal neuron firing activity constitutes a homeostatic or neuroprotective pathway, referred to as vasculo-neuronal coupling (VNC). In theory, VNC safeguards the brain from mismatches in metabolic demand and supply [11, 12]. Because VNC integrates communication among vascular, glial, and neuronal cells in the absence of neuronal stimulation, it provides an alternative stimulus framework for assessing the functional integrity of the NVU at rest. Here, we tested the hypothesis that chronic hypoperfusion uncouples VNC, establishing an early event that increases the vulnerability of the brain during mismatches in metabolic demand and supply.

Ischemia promotes ATP release and thereby contributes to an increase in adenosine levels [13, 14], highlighting the critical importance of adenosine’s role in neuroprotection. Adenosine signaling at the NVU is complex, reflecting the wide expression of diverse adenosine receptors, including A1Rs, A2_A_Rs, A2_B_Rs, and A3Rs, in multiple cell types and the fact that their activation can lead to varied responses. In neurons, adenosine functions as an inhibitory neuromodulator [15, 16], acting through presynaptic A1Rs to decrease Ca^2+^ influx and neurotransmitter release [17–19]. Postsynaptically, A1R activation hyperpolarizes neurons via G protein–coupled inwardly rectifying K^+^ channels [20]. Conversely, activation of A2_A_Rs in neurons is, in general, associated with increased excitability [21, 22]. A2_A_R and A2_B_R activation in vascular smooth muscle cells is associated with vasodilation [23, 24], whereas A1R activation, mainly at low-adenosine concentrations, can lead to vasoconstriction in some vascular beds, such as the renal [25], cerebral [26, 27], and pulmonary [28] circulation. Importantly, adenosine receptors are subject to potential desensitization or downregulation [29]. The functional role of A2_B_R in the brain is poorly understood. In response to ischemia, evidence support both a neuroprotective as well as a pro-excitatory role [30–32]. Taken together, these observations suggest that an imbalance in purinergic signaling at the NVU could promote activation of neuroprotective pathways (i.e., adenosine-induced neuromodulation) as well as pro-inflammatory (i.e., ATP-induced microglial activation) [13] and pro-excitatory pathways that contribute to neurodegeneration.

To shed light on the cellular mechanisms that lead to the progression of neurovascular dysfunction during chronic brain hypoperfusion, we used the bilateral common carotid artery stenosis (BCAS) model, a well-characterized model of vascular cognitive impairment. BCAS recapitulates many of the pathological hallmarks of vascular cognitive impairment, including hypoperfusion, white matter rarefaction, astrogliosis, microgliosis [33, 34], inflammation [35, 36], and cognitive dysfunction [35, 37]. Using an ex vivo pressurized brain slice preparation that enables assessment of the integral function of the NVU, we addressed how mild chronic hypoperfusion impacts vascular and neuronal function, and specifically tested the hypothesis that altered A1R-mediated signaling contributes to impaired vascular cognitive impairment in BCAS mice. Collectively, our findings support the conclusion that BCAS induces a reduction in parenchymal arteriolar tone, a process that is likely mediated by A1R dysregulation in vascular cells. Moreover, we demonstrate that this mechanism contributes to impaired flow of information between blood vessels and neurons under resting (basal) conditions.

## Materials and methods

### Animals

All experiments were conducted on 8–14-week-old male C57BL6 mice (Jackson Laboratories) following protocols approved by the animal care and use committee of Augusta University. Before experimentation, animals were housed in a room maintained at 20–22 °C with a 12-h:12-h light to dark cycle and provided ad libitum access to food and water.

### BCAS and laser Doppler perfusion imaging

Mice were anesthetized using 2% isoflurane, and an incision was performed to expose the skull. Baseline whole-brain CBF was subsequently measured using a Laser Doppler Perfusion Imager (LDPI; Perimed Periscan Pim 3), while maintaining a constant distance (10–10.9 cm) between the LDPI scanner and the mouse skull. Cortical blood flow was measured before the introduction of micro coils into the common carotid artery, performed immediately after, and in some mice, 14 days (14 d) or 28 days (28 d) after BCAS surgery. Blood flow values are reported as the percent changes in CBF from the values measured before BCAS surgery. Following baseline CBF recordings, a small incision was made to expose the common carotid artery. The artery was then carefully separated from the fascia and vagus nerve. Thereafter, the common carotid artery was gently lifted and a steel microcoil (0.08 mm string diameter, 0.18 mm inner diameter, 0.5 mm pitch, 2.5 mm total length; Wuxi Samini Spring and Sawane Spring) was twined around the artery immediately below the bifurcation of the internal and external carotid arteries. The procedure was then repeated on the contralateral side. Upon termination of BCAS surgery, the second CBF measurement was performed. Animals were allowed to fully recover until fully conscious and then given ad libitum access to food and water. Sham surgeries included all steps, excluding the addition of micro coils. Exclusion criteria: If the surgery was successful, based on a 30% or greater reduction in CBF with laser Doppler, animals were included in the study. This was the case for all animals used.

### Brain slice preparation

**Fig. 1:**
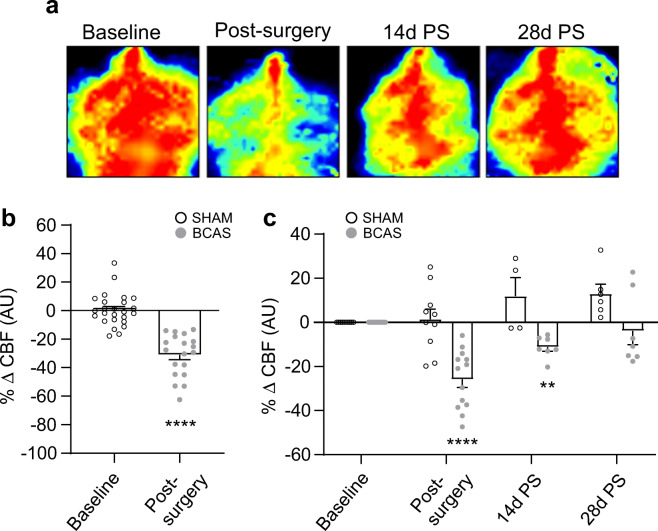
BCAS surgery-induced changes in CBF. **a** Temporal laser Doppler scan images at baseline (prior to BCAS surgery), immediately post-surgery (PS), and 14 or 28 days PS. **b** CBF measured in sham and BCAS mice at baseline and immediately PS (*n* = 25 for sham and *n* = 19 for BCAS). **c** CBF measured in sham and BCAS mice at baseline, immediately PS (*n* = 10 for sham and BCAS), and 14 days (*n* = 4/10 for sham/BCAS) or 28 days PS (*n* = 6/7 for sham/BCAS). CBF values are presented as means ± SEM, expressed as a percentage of baseline (^#^*P* < 0.05, sham versus baseline; ****P < 0.0001 BCAS versus baseline; mixed-effects model repeated-measures ANOVA followed by Dunnett’s multiple comparison test)

Following anesthesia with sodium pentobarbital, the brain was removed, cut into 250–300-μm-thick coronal slices using a vibratome (Leica VT 1200S; Leica Microsystems, Wetzlar, Germany), and placed in cold artificial cerebrospinal fluid (aCSF; 3 mM KCl, 120 mM NaCl, 1 mM MgCl_2_, 26 mM NaHCO_3_, 1.25 mM NaH_2_PO_4_, 10 mM glucose, 2 mM CaCl_2_, and 400 μM l-ascorbic acid; osmolarity, 300–305 mOsm), equilibrated with 95% O_2_/5% CO_2_. Slices were kept at room temperature (RT) in aCSF until transferred to the microscope chamber. All experiments were conducted at a chamber temperature of 33° ± 1 °C using a single-line solution heater (SH-27G; Warner Instruments, Hamden, CT) connected to a DC power supply (1735 A; BK Precision, Yorba Linda, CA) and continuously perfused with aCSF at a rate of 2–3 ml/min using a peristaltic pump (Miniplus 3; Gilson, Middleton, WI). Of note, mice used for brain slices (Fig. 1d) had not undergone multiple CBF measurements (Fig. 1c), other than that performed during the introduction of the coils at the time of surgery.

### Vessel cannulation

Arteriole cannulas (ID, 1.17 mm; OD, 1.50 mm; G150TF-3; Warner Instruments) were pulled with a P-97 micropipette puller (Sutter Instruments; Novato, CA), then beveled (KT brown type micropipette beveller BV-10; Sutter instrument) and mounted onto a micromanipulator. Prior to cannulation, the cannula resistance was determined from a flow-pressure curve, as previously reported [38]. Parenchymal arterioles were visualized using a × 60 Nikon (NIR Apo, × 60/1.0w, DIC N2, ∞/0 WD 2.8) objective equipped with infrared differential interference contrast (IR-DIC) optics. Luminal flow was controlled with a syringe pump (PHD 2000; Harvard Apparatus, Holliston, MA). A pressure transducer was placed immediately before the cannula for constant pressure monitoring (Servo Pump PS/200; Living System Instrumentation, Burlington, VT). The internal cannula solution consisted of 3 mM KCl, 135 mM NaCl, 1 mM MgCl_2_, 10 mM glucose, 10 mM HEPES, 2 mM CaCl_2_, and 1% albumin [39], with an osmolarity of 300–305 mOsm and pH 7.4 (adjusted with NaOH). The tip of the cannula was maneuvered toward the entrance of the parenchymal arteriole and slowly introduced into the vessel lumen [38]. Following cannula insertion, a second set of flow-pressure measurements was performed, and the total resistance (cannula + vascular bed) was calculated. Vascular resistance was determined by subtracting the cannula resistance from the total resistance. The calculated vascular resistance, determined from Ohm’s law, *Q* = Δ*P*/*R*, where *Q* is flow rate, Δ*P* is the difference in pressure, and *R* is resistance (in arbitrary units [AU]), was used to determine the flow rate (*Q*) required to reach intravascular pressures ranging from ~ 10 to 80 mmHg. Technical considerations and exclusion criteria: Successful experiments were characterized by low cannula resistance and a vascular contribution to total resistance greater than 45%. For sham, BCAS 14 d and BCAS 28 d experiment values for averaged cannula resistances were 21.9 ± 2 AU, 25.31 ± 3 AU, and 14.32 ± 1.1 AU; 46.02 ± 5.7 AU, 74.34 ± 16.3 AU, and 40.79 ± 10.5 AU for total resistances; and 24.12 ± 5, 49.03 ± 16.2 AU, and 51.65 ± 5.7 AU for vascular resistances, respectively. The vascular contribution to total resistance was 49.45 ± 3%, 58.22 ± 8%, and 51.65 ± 7% for sham, BCAS 14 d, and BCAS 28 d, respectively. When the vascular contribution is lower than 30%, arterioles are not able to develop tone.

#### Exclusion criteria

A total of six mice were excluded at the time of the BCAS surgery for a lack of CBF reduction. The minimum CBF reduction following the introduction of coils was 6%; this mouse was included in the study. A total of four vessels out of thirty-eight were excluded from the analysis. Exclusion included vessels that did not develop tone (2 shams, 1 BCAS 14), and one vessel from the sham group which showed a significant jump in the pressure reading indicative of the clogging of the cannula during the experiment.

### Vascular reactivity measurements

Studies of flow/pressure-induced myogenic tone in C57BL6 mice were performed in sham, BCAS 14 d, and 28 d post-surgery mice, as well as a set of naïve C57BL6 mice (Fig. 6b). Vascular diameter and lumen pressure values were recorded throughout the experiment at a frequency of 1 image/s using PCO camware and Clampex 9.2 software, respectively. Upon cannulation, the arteriole flow rate was set to induce a starting lumen pressure of ~ 30–40 mmHg (estimated physiological pressure for a parenchymal arteriole); these conditions were sustained until a plateau was established. After reaching a plateau, the flow rate was first decreased to reach a lumen pressure of ~ 10 mmHg (low pressure) and then stepwise increased up to a pressure of ~ 60–80 mmHg (high pressures). For CPT-induced vascular responses, parenchymal arterioles were first pressurized and equilibrated at 40 mmHg and then perfused with the A1R antagonist CPT (1 μM). At the end of the experiment, slices were perfused with zero Ca^2+^ aCSF containing 100 μM papaverine to obtain maximum diameter (100%) at each intravascular pressure (~ 10–120 mmHg) used for each protocol. Diameter values are expressed as %tone relative to the average maximum diameter at each flow rate used. For percent relaxation, tone was calculated relative to baseline tone.

### Electrophysiology

Whole-cell currents were obtained using a Multiclamp 700B amplifier (Axon Instruments, Foster City, CA). Patch pipettes were made from thin-walled borosilicate glass (OD, 1.5 mm; ID, 0.86 mm; Sutter instrument BF150-86-7.5) and pulled using a P-97 puller (Sutter Instruments) to a resistance of 4–6 MΩ. The internal solution consisted of 130 mM K-gluconate, 10 mM HEPES, 10 mM BAPTA, 10 mM KCl, 0.9 mM MgCl_2_, 4 mM Mg_2_ATP, 0.3 mM Na_2_GTP, and 20 mM phosphocreatine, with an osmolarity of 291–295 mOsm and pH 7.2 (adjusted with KOH). A square-pulse protocol was used to determine whether the recording neuron showed spike frequency adaptation, indicative of a pyramidal neuron, as previously described [40]. For current-clamp mode recordings, and to generate neuronal action potentials (AP), neurons were brought to near spike threshold (approximately − 45 mV) by injecting a depolarizing DC current and holding it until a stable AP frequency was established. Current signals were filtered with a 1-kHz low-pass filter and digitized at 10 kHz using a Digidata 1322A acquisition system (Axon Instruments). Input-output function was assessed by subjecting patched neurons to depolarizing steps of increasing magnitude (0–140 pA) and plotting the number of evoked spikes as a function of depolarizing steps. Miniature GABA inhibitory postsynaptic currents (mIPSCs) and glutamate excitatory postsynaptic currents (mEPSCs) were simultaneously recorded at a holding potential of − 40 mV in the presence of 1 μM tetrodotoxin (TTX). IPSCs and EPSCs were distinguished by their outward and inward current polarity, respectively [41]. Voltage output was digitized at 16-bit resolution and 10 kHz and filtered at 2 kHz. pClamp10.6 (Axon Instruments) was used for data acquisition and storage. Only neurons with a stable baseline firing activity following depolarizing DC current injections were used.

### Reverse transcription polymerase chain reaction and quantitative real-time PCR from brain region-specific tissue punches

RNA extraction and isolation were performed using the miRNAeasy Mini kit (Qiagen, Cat. No. 217004) and the QIAzol Lysis Reagent (Qiagen, Mat. No. 1023537) according to the manufacturer protocol. One hundred-micrometer-thick tissue sections were obtained using a cryostat (− 20 °C, Leica, CM3050S) and punches from corpus callosum (Bregma 1.1 mm to Bregma − 0.1 mm), somatosensory cortex S1BF (Bregma − 0.4 mm to Bregma − 1.5 mm), and dorsal hippocampus (Bregma − 1.2 mm to Bregma − 2.6 mm) were collected bilaterally using a 0.75-mm tissue puncher, and samples were kept on dry ice until the RNA extraction procedure. The precise anatomical location and coordinates are highlighted in Fig. 7. RNA concentration was measured using NanoDrop One (Thermo Scientific) and was in the range of 55–135 ng/μl prior to cDNA synthesis. cDNA synthesis was performed using the iScript™ gDNA Clear cDNA Synthesis Kit (BIO RAD, Cat. No. 1725035) and the SimpliAmp Thermal Cycler (Applied Biosystems, Thermo Fisher Scientific) according to the manufacturer protocol. qPCR was conducted using the Roche Lightcycler96 with the default 45-step amplification protocol and the following 10× QuantiTect primers (diluted in 1.1 mL TE pH 8.0, final concentration: 200 nM) purchased from Qiagen: Adenosine receptor 1a (QT00301119), Adenosine receptor 2b (QT00257558) and Actin-Beta (reference gene, QT01136772). For the calculation of A1/A2 ratios, we used the LightCycler96 software and its built-in algorithm for relative quantification (using the standard formula (±)2^ΔCq^) and compared A1 relative to A2b, which served as the referenced gene. All individual qPCR reactions (brain region, primer, and condition) were duplicated, and average values were calculated and used for statistical analysis.

### Data analysis

Electrophysiology data were analyzed using Clampfit 10.6 (Axon Instruments). Firing discharge was recorded in current-clamp mode. The mean frequency was obtained before, during, and after bath application of drug (adenosine) or stimulus (increased intravascular pressure). Cell capacitance was calculated by integrating the area under the transient capacitive phase of a 5-mV depolarizing step pulse (voltage-clamp mode). For mIPSCs and mEPSCs, detection thresholds were set at 5 and 8 pA, respectively. PSC frequency and waveform parameters were analyzed using Mini Analysis software (Synaptosoft, Leonia, NJ). The charge transfer (*Q*) was determined from the area under the PSC waveform. The mean PSC current was calculated as the *Q* of the averaged PSC multiplied by the mean PSC frequency [41].

GraphPad Prism 8 software (GraphPad Software, La Jolla, CA) was used for all statistical analyses. Values are expressed as means ± S.E.M. Differences between groups were determined using one- or two-way repeated-measures analysis of variance (ANOVA) with corresponding multiple comparison post hoc tests, as specified in figure legends. Statistical significance was accepted at the 95% (*P* < 0.05) confidence level, and individual *P* values, determined from multiple comparison tests, are indicated in the text and figure legends.

## Results

### BCAS-induced changes in cerebral perfusion

CBF measurements were made before BCAS surgery (baseline), immediately after surgery, and 14 d or 28 d post-surgery. Consistent with previous studies [42], introduction of micro coils into the common carotid artery significantly decreased CBF immediately after surgery (31.05% ± 3.4% reduction, *P* < 0.0001), Fig. 1a, b. In a few mice, CBF was measured immediately after surgery (25.8 ± 3.7 reduction, *P* < 0.0001) and then 14 and/or 28 days post-surgery. Fourteen days post-surgery, blood flow had partially recovered (11.2% ± 1.5% reduction) but remained significantly lower than at baseline (*P* = 0.001). In contrast, CBF measured in 28 d post-surgery mice (3.9% ± 6.3% reduction) was not significantly reduced compared with that at baseline (*P* = 0.8) (Fig. 1c).

### Parenchymal arterioles from BCAS mice lose reactivity to intravascular pressure

**Fig. 2:**
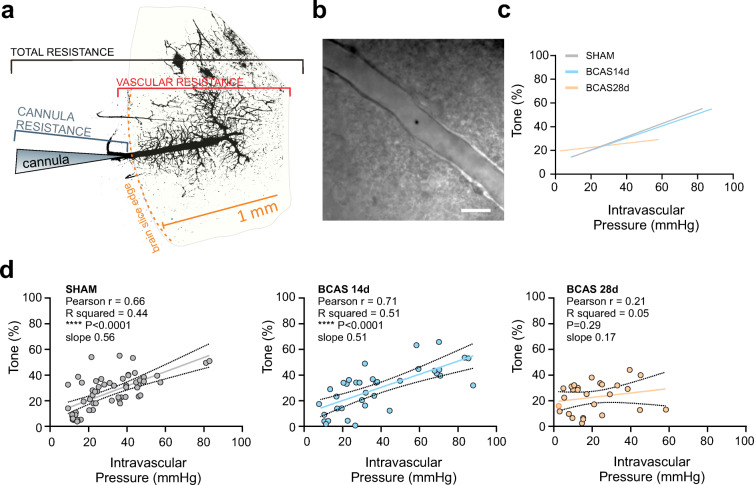
BCAS-driven changes in parenchymal arteriolar tone. **a** Components of the brain slice cannulation technique that define the elements that contribute to the total resistance of the system: cannula resistance and vascular resistance (resistance of the perfused network downstream from the cannulated parenchymal arteriole). The binary image corresponds to a cannulated arteriole labeled with FITC (post-experiment) to define the downstream vascular network. **b** Representative differential interference contrast image of a cannulated parenchymal arteriole in a brain slice. **c** Averaged linear regression line for the intravascular pressure-versus-tone (%) relationship for sham (*n* = 15 vessels from 11 mice), BCAS 14 d (*n* = 9 vessels from 6 mice), and BCAS 28 d (*n* = 10 vessels from 8 mice) groups. **d** Intravascular pressure-versus-tone (%) responses for each experimental group shown in **c**

Using an ex vivo brain slice preparation that incorporates flow and pressure within a parenchymal arteriole [38], we measured pressure-evoked (myogenic) changes in parenchymal arteriolar tone in C57BL6 sham and BCAS 14 d and 28 d post-surgery mice. We identified and cannulated a parenchymal arteriole (Fig. 2a, b) and used the resistance of the perfused vascular network to determine the flow rate needed to achieve intravascular pressure ranges between 10 and 80 mmHg, as described in “Materials and methods.” Following the development of tone at an estimated physiological pressure of ~ 30 mmHg, flow rates were increased to evoke stepwise increases in pressure to final values of ~ 60–80 mmHg. Raising intravascular flow/pressure increased parenchymal arteriolar tone to a similar extent in both sham and BCAS 14 d mice (Fig. 2c, d, left and middle panels). However, myogenic responses were reduced in the BCAS 28 d group, in which a leftward shift in the pressure-tone relationship and a loss of pressure-tone correlation were observed (Fig. 2d, right panel). To better illustrate the decrease in the slope of the relationship in BCAS 28 d mice, we show superimposed regression lines of pressure-tone relationships obtained in each group in Fig. 2c.

### Impaired VNC in BCAS mice

Lack of tone would suggest a potential compensatory mechanism in BCAS mice. However, what impact BCAS has on neuronal function is poorly understood. We previously reported that myogenic-induced vasoconstriction triggers a cascade of signaling events in healthy rats and mice that leads to inhibition of cortical pyramidal neuron firing activity [11]. This putative neuroprotective VNC process safeguards the brain during decreases in CBF and from hyperperfusion when pressure is high. Here, we sought to assess the impact of BCAS on vessel-to-neuronal communication. Using electrophysiological recordings in both voltage-clamp and current-clamp mode, we monitored the passive and active membrane properties of cortical pyramidal neurons in sham and BCAS mice. No differences among sham (*n* = 57), BCAS 14 d (*n* = 46), and BCAS 28 d (*n* = 55) groups were observed for resting membrane potential (− 69.81 ± 0.6, − 70.34 ± 0.6, and − 70.48 ± 0.7 mV, respectively), input resistance (166.2 ± 8.1, 152.0 ± 9.5, and 154.2 ± 7.7 MΩ, respectively), or cell capacitance (43.6 ± 1.7, 40.2 ± 1.5, and 41.3 ± 1.0 pF, respectively) (Supplementary Fig. 1), although a tendency toward lower input resistance was observed in BCAS 14 d and 28 d mice. We also measured neuronal action potential (AP) frequency in response to depolarizing current steps of increasing magnitude (0–140 pA) (i.e., input-output function). While the number of evoked APs significantly increased as a function of stimulus magnitude (*F* = 32.4, *P* < 0.0001, 2-way repeated-measures ANOVA), no differences among experimental groups were observed (*F* = 0.4, *P* = 0.3) (Supplementary Fig. 1). These results indicate that chronic hypoperfusion does not change the basic membrane properties or input-output function of cortical pyramidal neurons, at least in this model and within the time frame examined.

**Fig. 3:**
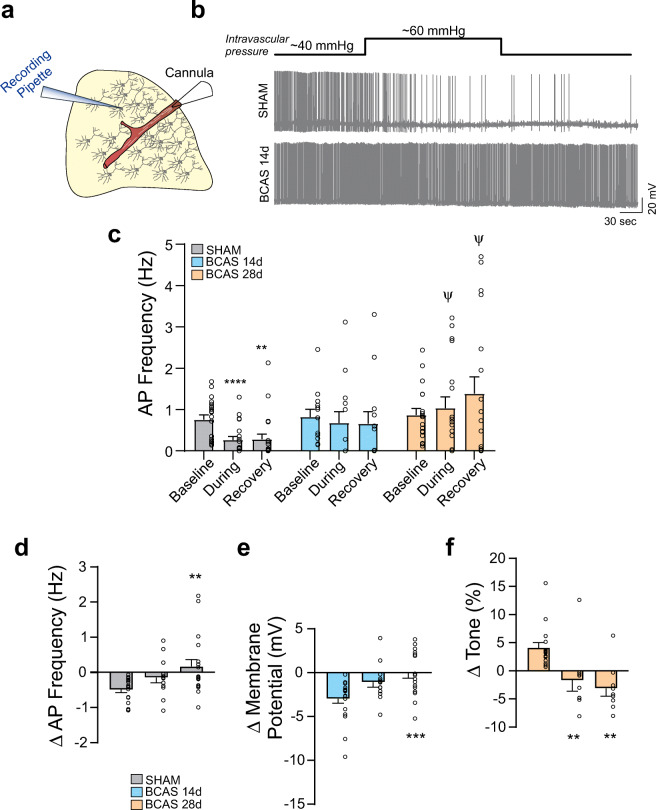
BCAS-induced impairments in VNC. **a** Illustration of a pressurized brain slice preparation showing the position of the cannulated arteriole relative to the recorded pyramidal neuron (typically < 100 μm). **b** Representative membrane potential traces showing the response of a pyramidal neuron to an increase in intravascular pressure from ~ 40 to 60 mmHg. **c** Summary data showing AP frequency in response to increases in intravascular pressure within a nearby parenchymal arteriole (sham, *n* = 20 neurons from 6 mice; BCAS 14 d, *n* = 13 neurons from 3 mice; BCAS 28 d, *n* = 18 neurons from 4 mice). Data are presented as means ± SEM (**P < 0.01 versus baseline for within-group comparisons; ^ψ^*P* < 0.05 versus sham for between-group comparisons; two-way repeated-measures ANOVA followed by Dunnett’s multiple comparison test). **d** Summary data showing the change (Δ) in AP frequency, **e** membrane potential (mV), and **f** tone in response to an increase in intravascular pressure within a nearby parenchymal arteriole in sham (*n* = 16 arterioles from 6 mice), BCAS 14 d (*n* = 9 arterioles from 3 mice) and BCAS 28 d (*n* = 9 arterioles from 4 mice) groups. Data are presented as means ± SEM (**P < 0.01, ***P < 0.001 versus baseline; two-way repeated-measures ANOVA followed by Kruskal-Wallis test)

We then measured the VNC response, namely pyramidal neuronal responses to myogenic constriction of parenchymal arterioles. To evoke APs, we depolarized silent neurons with DC current injection until a stable firing activity was achieved. Firing frequency was then recorded before and during a step increase in intravascular pressure from ~ 40 to ~ 60 mmHg (Fig. 3b). As previously reported [11], increases in intravascular flow/pressure significantly inhibited pyramidal neuron firing activity in sham mice (*P* < 0.0001). Strikingly, inhibitory responses were absent in BCAS 14 d (*P* = 0.52) and BCAS 28 d (*P* = 0.62) mice (Fig. 3c). In fact, an examination of the *delta* response for each group revealed that pressure-induced inhibition was converted to a pressure-induced increase in firing activity (*P* = 0.006) and a loss of membrane hyperpolarization (*P* = 0.0009) in BCAS 28 d mice (Fig. 3d, e). Consistent with a lack of myogenic responses, the change in tone evoked during the pressure stimulus was significantly smaller in BCAS 14 d (*P* = 0.003) and BCAS 28 d (*P* = 0.001) mice (Fig. 3f). These data support the idea that compromised myogenic constriction and the consequently altered biomechanics at the NVU constitute a critical step in vessel-to-neuronal signaling and suggest that BCAS decouples this communication modality.

### Adenosine-induced cortical pyramidal neuron responses in sham and BCAS mice

**Fig. 4:**
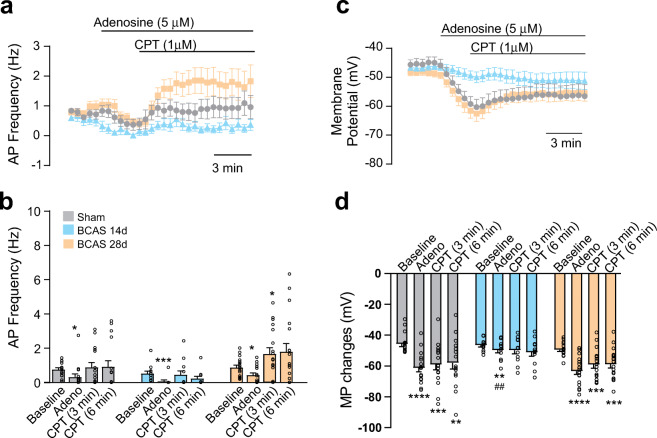
Adenosine-evoked changes in pyramidal neuron activity in sham and BCAS mice. **a** Average AP frequency in response to bath-applied adenosine followed by the A_1_R blocker, CPT. **b** Summary data showing AP frequency in sham (*n* = 16 neurons from 5 mice), BCAS 14 d (*n* = 12 neurons from 4 mice), and BCAS 28 d (*n* = 18 neurons from 4 mice) groups. **c** Average membrane potential (mV) changes in response to bath-applied adenosine followed by the A_1_R blocker, CPT (same neurons as in **a**, **b**). **d** Summary data showing membrane potential changes in sham, BCAS 14 d, and BCAS 28 d groups (same neurons as in **a**, **b**). Data are presented as means ± SEM (*P < 0.05, ***P < 0.001 versus baseline; two-way repeated-measures ANOVA followed by Dunnett’s multiple comparison test)

We previously reported that VNC is mediated, in part, by activation of A1Rs [11]. Because adenosine levels are increased following ischemia [43], constituting a critical neuroprotective pathway [44], we determined the effect of adenosine on pyramidal neuron firing activity in sham, BCAS 14 d and BCAS 28 d mice, measuring AP frequency. First, to evoke APs, we depolarized silent neurons with DC current injection until a stable firing activity was achieved, and we then bath-applied adenosine (5 μM). To determine the reversibility of the response, we bath-applied CPT (1 μM), a specific A1R blocker, in the continuous presence of adenosine. Adenosine affected AP frequency in all groups, significantly decreasing it in sham (*P* = 0.03), BCAS 14 d (*P* = 0.001), and BCAS 28 d (*P* = 0.02) mice (Fig. 4a, b). Likewise, adenosine induced a significant membrane potential hyperpolarization in all groups including sham (*P* = 0.0001), BCAS 14 d, (*P* = 0.003) and BCAS 28 d (*P* < 0.0001) (Fig. 4c, d). Notably, CPT reversed the adenosine-induced decrease in AP frequency in sham and BCAS mice; BCAS 28 d mice also exhibited a small overshoot in the AP response that was significant (*P* = 0.02) 3 min after bath application of CPT. Unlike its reversible effect in sham and BCAS 28 d, CPT failed to reverse the membrane potential hyperpolarization in BCAS 14 d (*P* = 0.11 and *P* = 0.05 for 3 and 6 min after bath application). Together, these data support the conclusion that adenosine-evoked inhibition of neuronal firing is still present in BCAS mice. However, the effect of adenosine on membrane potential was significantly blunted in BCAS 14 d (*P* = 0.002 versus sham group) and reversed by 28 d post-surgery.

### BCAS does not affect the overall excitatory/inhibitory synaptic balance in cortical pyramidal neurons

**Fig. 5:**
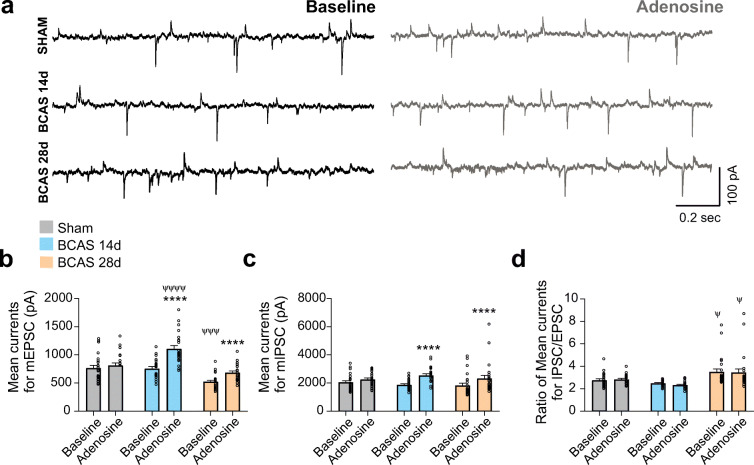
Adenosine-evoked changes in pyramidal neuron activity: excitatory and inhibitory currents. **a** Representative traces of miniature EPSCs and IPSCs at baseline (left) and in the same neuron in response to bath-applied adenosine (right) under voltage-clamp conditions. **b** Average mean EPSC currents, **c** IPSC currents, and **d** IPSC/EPSC ratios for all groups (sham, *n* = 24 neurons from 4 mice; BCAS 14 d, *n* = 24 neurons from 4 mice; BCAS 28 d, *n* = 24 neurons from 4 mice). Data are presented as means ± SEM (****P < 0.0001 for within-group comparisons; two-way repeated-measures ANOVA followed by Sidak’s test; ^ψψψ^*P* = 0.0001, ^ψ^*P* < 0.05 for between-group comparisons; two-way repeated-measures ANOVA followed by or Dunnett’s multiple comparison test)

Given that adenosine can act both pre- and postsynaptically to modulate both excitatory and inhibitory synaptic function [45, 46], we assessed the overall inhibitory/excitatory synaptic balance in cortical pyramidal neurons from sham and BCAS mice. To this end, we bath-applied adenosine and simultaneously monitored miniature excitatory postsynaptic currents (mEPSCs) and inhibitory postsynaptic currents (mIPSCs) in voltage-clamped cortical neurons. We then measured and calculated mean excitatory and inhibitory currents, which reflect the mean integrated synaptic current over time (see “Materials and methods”). As an estimate of the excitatory/inhibitory balance in each condition, we compared the ratio of mean inhibitory to mean excitatory synaptic input activities (IPSC/EPSC) (Fig. 5b–d).

Overall, our results show that adenosine did not change mean EPSC (Fig. 5b) or IPSC currents (Fig. 5c) in sham mice; thus, the inhibitory/excitatory balance, which was dominated by inhibition (i.e., 2/1 ratio), was unaltered (Fig. 5d). On the other hand, adenosine significantly increased both mean IPSC and EPSC currents in BCAS 14 d and 28 d mice (*P* < 0.0001 for all comparisons). However, this simultaneous increase in IPSCs and EPSCs again resulted in an unaltered excitatory/inhibitory balance in BCAS mice. Notably, the degree of baseline EPSC, but not IPSC, activity in BCAS 28 d mice was significantly reduced compared with shams (*P* = 0.0001), resulting in a slight, but significant increase in the inhibitory/excitatory balance in this group compared with the sham group (*P* = 0.01, for baseline) (Fig. 5d). These results indicate that differences in the inhibitory/excitatory balance, which influence cortical pyramidal neurons or their responsiveness to adenosine, are not major contributing factors to the lack of a VNC response in BCAS 28 d mice.

### Reduced adenosine-evoked vascular responses in BCAS mice

**Fig. 6:**
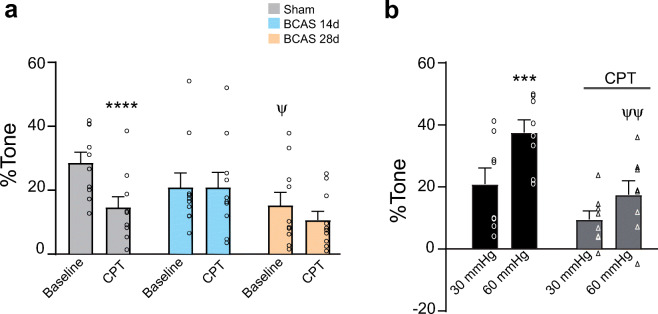
Adenosine-evoked parenchymal arteriole responses. **a** Effects of A_1_R blockade with CPT on parenchymal arteriolar tone (sham, *n* = 10 vessels from 6 mice; BCAS 14 d, *n* = 9 vessels from 5 mice; BCAS 28 d, *n* = 10 vessels from 4 mice). **b** Parenchymal arteriole responses to an increase in intravascular pressure in the absence and presence of CPT (*n* = 8 vessels from 4 mice). Data are presented as means ± SEM (*P < 0.05, **P < 0.01, ***P < 0.001 ****P < 0.0001 for within-group comparisons, two-way repeated-measures ANOVA followed by Sidak’st test; ^ψψ^*P* < 0.005 for between-group comparisons, two-way repeated-measures ANOVA followed by Dunnett’s multiple comparison test)

The authors of a recent study using a model of unilateral vessel occlusion suggested that metabolic demands are sustained through active mechanisms that maintain constant blood flow in the brain, although the signaling pathways involved remained unknown [47, 48]. Thus, altered adenosine signaling could alternatively impair adaptive vascular responses to chronic ischemia. In arterioles, A2_A_R and A2_B_R activation is associated with vasodilation, whereas A1R activation mediates vasoconstriction [49, 50]. To determine if the reduced parenchymal arteriolar tone we found in BCAS mice resulted from altered adenosine signaling at the vascular level, we measured baseline tone in the presence of the A1R antagonist CPT. Parenchymal arterioles were pressurized to ~ 40 mmHg and then perfused with CPT. In the presence of CPT, parenchymal arterioles from sham mice exhibited a significant reduction in baseline tone (*P* < 0.0001). In contrast, CPT had no significant effect on baseline tone in BCAS 14 d (*P* > 0.99) or BCAS 28 d (*P* = 0.22) mice (Fig. 6a). To determine if A1R activation participates in myogenic reactivity, we measured the myogenic response of parenchymal arterioles from naïve C57BL6 mice in the presence and absence of CPT during an intravascular pressure challenge. Increasing intravascular pressure from 30 to 60 mmHg significantly increased tone (*P* = 0.0008). Arterioles were then returned to 30 mmHg, and the brain slice was perfused with CPT. CPT alone produced a trend toward reduced arteriolar tone that did not reach significance, but it did significantly blunt the ability of arterioles to respond to the same flow rate used to increased intraluminal pressure to 60 mmHg (*P* = 0.09). These data support the conclusion that the contribution of A1Rs to baseline tone is impaired in BCAS mice, and that these adenosine receptors constitute key molecular contributors to the myogenic response of cortical parenchymal arterioles (Fig. 6b).

### Reduced mRNA A1R to A2_B_R ratio in BCAS 14 d

**Fig. 7:**
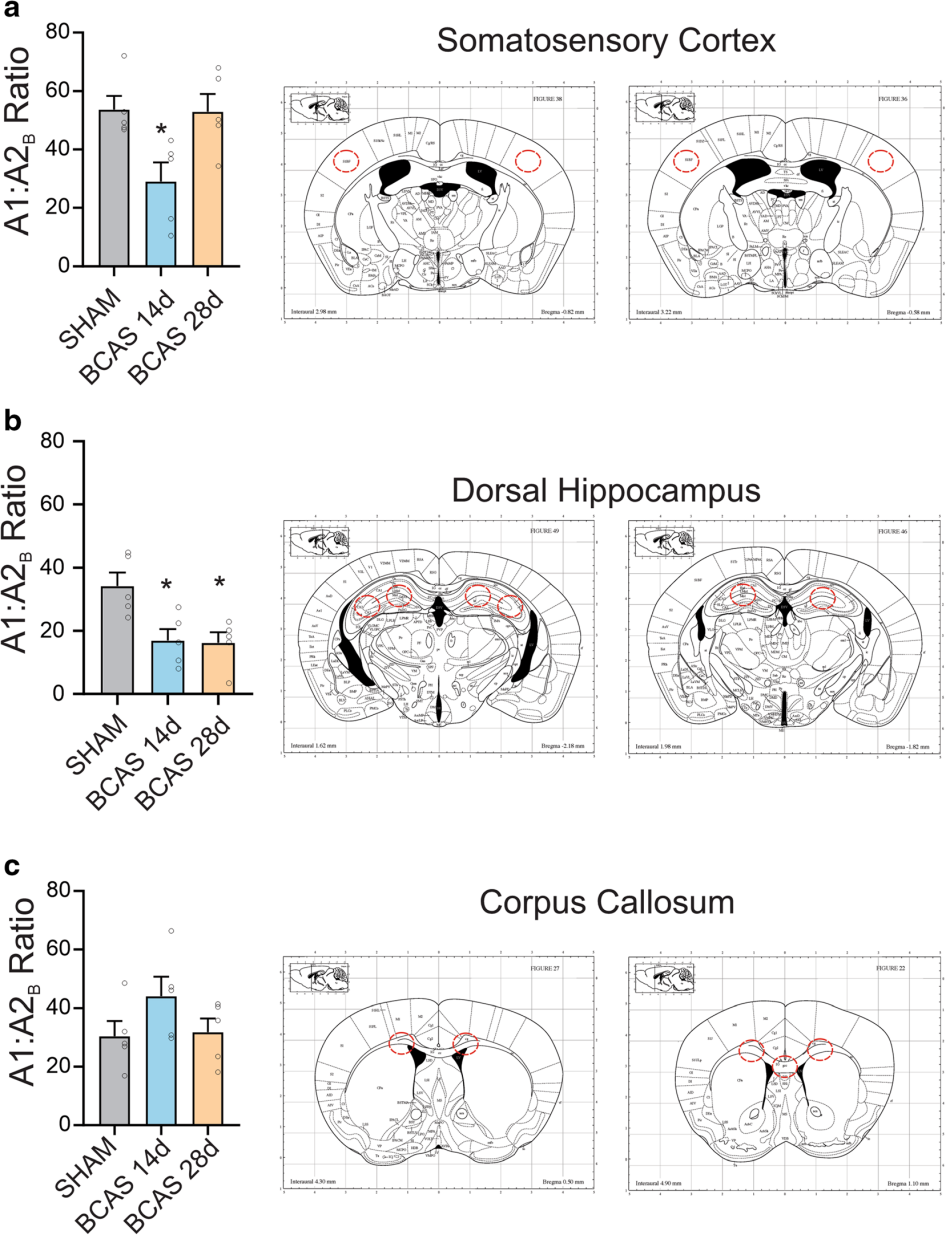
Reduced A1R to A2_B_R mRNA ratio in BCAS 14 d. Summary of the mean fold change in mRNA expression of A1R relative to A2_B_R in Sham, BCAS 14 d, and BCAS 28 d groups (*n* = 5 mice per group). Schematics to the right show the corresponding brain regions from where brain punches were obtained (modified from Paxinos mouse atlas). Data are presented as mean ± SEM (*P < 0.05 versus sham, one-way ANOVA followed by Tukey’s multiple comparison test)

The data above support dynamic changes in the impact of adenosine receptor function on different components of the NVU. To determine whether changes in the relative expression of these opposing acting receptors changed during the course of BCAS, we used qPCR to measure the mRNA expression ratio of A1R to A2_B_R in various brain regions, including the somatosensory cortex, dorsal hippocampus (DH), and the corpus callosum. In somatosensory cortex, mRNA levels from BCAS 14 d, when compared to shams, showed a significant reduction in A1R to A2_B_R expression (*P* = 0.03). This reduction was significantly reversed (*P* = 0.03 versus BCAS 14 d), reaching comparable control levels by day 28 post-surgery. In the hippocampus, a highly vulnerable brain region to ischemia, the A1R to A2_B_R ratio remained significantly reduced (*P* = 0.02, *P* = 0.01 for BCAS 14 d and BCAS 28 d versus sham, respectively). No significant changes were observed in the corpus callosum (Fig. 7). Together, these data support dynamic changes in the levels of A1 and A2_B_ receptors following BCAS surgery, in which a diminished A1 to A2_B_ ratio could contribute to a blunted A1R vasoconstriction at the arteriole level and blunted VNC at the neuronal level.

## Discussion

Our study demonstrates that mild (~ 30%) chronic hypoperfusion of the brain significantly alters the functional integrity of the cortical NVU. Although surface cerebral perfusion recovered by day 28 post-BCAS surgery, parenchymal arterioles from BCAS mice showed a progressive decrease in tone. Moreover, vessel-to-neuron communication was diminished, suggestive of impaired VNC. This decoupling of parenchymal arteriole-to-neuronal communication involves altered A1R-mediated responses.

### BCAS-induced decreased parenchymal arteriole tone

The decrease in vascular tone in BCAS 28 d mice is consistent with previous reports using alternative models of chronic hypoperfusion. Using unilateral common carotid artery occlusion in rats, Cipolla’s group showed diminished parenchymal arteriolar tone after 4 weeks of occlusion [47, 48]. The authors postulated that the decrease in tone was an adaptive mechanism involving activation of endothelial TRPV4 channels that serves to restore perfusion to the brain [47]. The potential contribution of these channels to the regulation of arterial tone via endothelial TRPV4 cannot be ruled out, as they are critical for endothelial-mediated dilation [51–55], known to be impaired by brain ischemia [56, 57], and are contributors to angiogenesis and neovascularization [58].

**Fig. 8:**
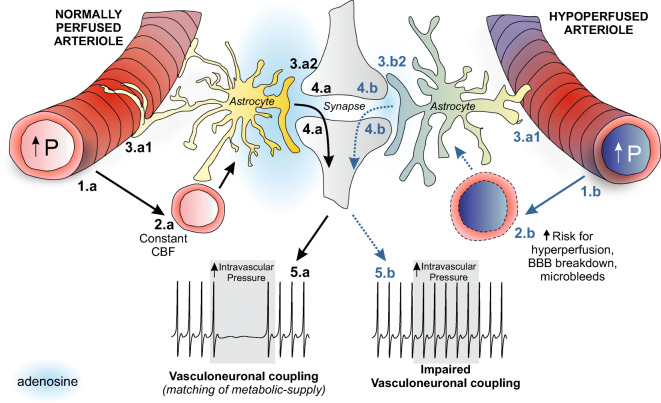
Working model for impaired vasculo-neuronal coupling in BCAS. We predict that in a normally perfused neurovascular unit (NVU), an increase in intravascular pressure (1.a) will cause parenchymal arteriolar constriction. The resulting biomechanical stimulation at the NVU (i.e., mediated via increased tone) triggers an increase in astrocyte Ca^2+^ (2.a) and release of ATP/adenosine both at the gliovascular interface (3.a1) as well as the synapse (3.a2). A1R activation on parenchymal arterioles contributes to the maintenance of myogenic constriction. At the synapse, A1R activation causes suppression of neuronal activity (4.a). Thus, the increased parenchymal arteriolar constriction, which would decrease downstream blood flow, suppresses neuronal activity to keep metabolic demands and supply in balance (5.a). On the other hand, in a hypoperfused NVU, the increase in intravascular pressure fails to induce myogenic constriction and consequent biomechanical stimulation on astrocytes (1.b), increasing the risk for hyperperfusion, breakdown of the blood-brain barrier (BBB), and microbleeds. Furthermore, blunted biomechanics at the NVU (i.e., due to lack of tone) (2.b) along with a potential decrease in A1R-mediated signaling (3b1, 3b2) fails to decrease neuronal activity (4.b), increasing the risk for unbalanced metabolic demands and supply (5.b)

While not addressed in this study, ischemia could profoundly affect the function of astrocytes, which in turn modulate resting arteriolar tone [12, 59, 60] as well as neuronal function. Ischemia induces the release of adenosine from both astrocytes and neurons [13, 15, 61, 62]. Notably, TRPV4 ion channels have been implicated in the release of ATP [63, 64], raising the possibility that, in astrocytes, reduced TRPV4 channel activation results in diminished ATP release. Based on our previous findings that myogenic constriction activates TRPV4 ion channels in astrocyte endfeet [12], and that A1R activation contributes to arteriole tone, we propose that diminished myogenic responses in BCAS reduced biomechanically activated endfeet TRPV4 channels and, consequently, reduced adenosine availability at the gliovascular interface (Fig. 8).

In addition, because the perfused arteriole in the pressurized brain slice preparation includes a downstream capillary network (Fig. 2a), an additional contributor to the reduction in vascular resistance could be pericyte dropout. A decrease in pericyte coverage around capillaries, as previously reported in ischemia and vessel remodeling [65, 66], might decrease the ability of the perfused vascular network to sustain intravascular pressure, contributing to an overall loss of resistance. While this is an inherent limitation of the technique, the same limitation could apply in an in vivo setting.

Importantly, the diminished parenchymal arteriolar tone persisted even after CBF was ostensibly restored, raising the question of whether the ischemic period permanently altered the ability of these arterioles to respond to increases in intravascular pressure, in turn compromising critical physiological processes such as autoregulation. If so, this would explain the leftward shift in the cerebral autoregulation curve reported with chronic hypoperfusion and the increased vulnerability of the cerebral microcirculation to pressure fluctuations, in particular, high pressure-induced hyperperfusion [67].

### BCAS-induced decoupling of vascular to neuronal communication

Neurovascular coupling studies assess the functional integrity of the NVU in the context of a stimulus originating from active neurons (i.e., neuron-to-vascular signaling). During this process, the activity-dependent release of vasodilatory signals overrides constitutive mechanisms that maintain constant flow. Thus, while the neurovascular coupling response is critical for determining whether neurons receive needed energy supplies during enhanced activity, it does not provide a framework for understanding mechanisms that set baseline CBF. However, considering that neurovascular coupling is largely reported as a percent change in CBF from baseline, understanding the cellular mechanisms governing baseline tone is critical as these processes could impact the waveform (e.g., onset, amplitude, duration) of the neurovascular coupling response. Here, we used an experimental paradigm that not only evaluates the cellular mechanisms underlying baseline tone but also the flow of information at the NVU under circumstances in which the signal (or stimulus) is of vascular origin, such as intravascular pressure and/or flow, a phenomenon termed vasculo-neuronal coupling (VNC) [11, 68]. A major finding of this study is that BCAS mice showed reduced parenchymal arteriole constriction-induced inhibition of pyramidal neuron firing activity. Adenosine is a crucial neuroprotective player during ischemia [15, 44, 69, 70], and in the healthy brain, we have identified adenosine, acting via A1Rs, as a critical mediator of VNC. Specifically, A1R blockade prevents the neuronal inhibition triggered by arteriole constriction [11]. Still, given that adenosine can act at all levels of the NVU, the key cellular targets that mediate adenosine effects on VNC have remained unknown.

Based on these findings, we sought to determine whether altered adenosine signaling contributes to impaired VNC in BCAS mice and attempted to identify the possible cellular target that mediates this effect. We first asked whether mild chronic hypoperfusion alters the neuronal response to adenosine. In BCAS mice, basic neuronal properties were not affected. However, while adenosine-evoked inhibition of neuronal AP frequency in all groups, a significant reduction in the adenosine-induced neuronal hyperpolarization was observed in BCAS 14 d. At the synaptic level, the data did not support significant adenosine-evoked modulation of the excitatory/inhibitory synaptic balance. Adenosine induced both IPSC and EPSC responses in BCAS mice, and because these changes were proportional, they caused no significant differences in the overall excitatory/inhibitory synaptic balance. A significant increase in the IPSC/EPSC mean current ratio was observed at baseline in the BCAS 28 d group, with the excitatory/inhibitory balance shifted slightly to an even stronger inhibitory predominance compared with that in sham mice. Together with reduced proportional mRNA expression of A1R relative to A2_B_R in BCAS 14 d, these data are suggestive of a reduction in adenosine-induced neuronal suppression.

At low-adenosine concentrations, A1R activation can evoke vessel constriction. Here we show that parenchymal arterioles exhibited altered responses to adenosine in BCAS mice. Our findings indicate that adenosine, acting via A1R activation, plays a role in the regulation of baseline tone; importantly, this function is lost in BCAS mice. Moreover, we showed that A1Rs are important modulators of the myogenic response to increases in pressure. Taken together with our previous finding that A1R blockade prevents inhibition of neuronal firing following an increase in intravascular pressure, our current data provide strong support for the idea that blunted A1R signaling is a key mechanism that contributes to impaired VNC in BCAS mice. In this context, it is possible that chronic ischemia desensitizes or downregulates A1R expression in blood vessels, as previously reported in the pulmonary circulation [28]. This phenomenon has been proposed as one of the mechanisms underlying the failure of A1R activators to provide therapeutic benefits [29].

Our quantitative PCR study showing a reduced A1R mRNA expression, relative to A2_B_R, in both cortex and hippocampus is consistent with this notion. While the role of A2_B_R is poorly understood, these receptors have less affinity to adenosine, are scarcely but uniformly expressed in brain [71], and have been implicated in ischemia [30–32]. We show that A2_B_R were also dynamically altered by BCAS supporting the need to further investigate their role in mild chronic hypoperfusion.

### Limitations of the BCAS model

The BCAS model recapitulates several pathophysiological hallmarks of chronic hypoperfusion that lead to cognitive impairment [72]. Studies using this model have reported an array of complications such as behavioral deficits [73], inflammation, white matter damage, significant decreases in cognitive performance [35, 74], altered arteriolar tone [47], endothelial dysfunction [75, 76], impaired glymphatic function [77], and loss of BBB integrity [78] even 1 month after surgery, establishing BCAS as a valuable model for dissecting the cellular mechanisms through which chronic hypoperfusion acts to cause neurovascular dysfunction. A limitation of the model, however, is the abrupt reduction in CBF which is then followed by a progressive recovery [79]. Thus, while the model recapitulates a number of conditions observed in individuals with vascular cognitive impairment, it would be important to compare vascular and neuronal responses in a model that leads to a progressive reduction in CBF (carotid artery stenosis). To this end, the alternative ameroid constrictor model [80–84] should be considered. Nonetheless, the BCAS model provides insights on the impact transient ischemia (7–14 days) has on neurovascular function as well as on the adaptive and/or recovery processes of the brain when challenged with a transient rather than progressive insult.

## Conclusions

Together, our findings suggest functional alterations at the NVU during BCAS (Fig. 8). Specifically, we demonstrated that BCAS induces a progressive decrease in parenchymal arteriolar tone and reactivity to adenosine. While the decreased tone may underlie an essential adaptive process for restoring perfusion to the brain, its neuroprotective function may be short-lived as this putative adaptive response was associated with reduced vascular reactivity to pressure. Importantly, this vascular dysfunction would increase the vulnerability of the cerebral microcirculation to pressure fluctuation-induced insults, such as BBB breakdown, ischemia, and microbleeds, all of which contribute to cognitive decline. Thus, we propose BCAS-induced changes in vascular function disrupted the functional integrity of the NVU. Because cortical pyramidal neuron function was less affected in BCAS, these results support the conclusion that early vascular dysfunction, via altered A1R signaling, is a critical event in the decoupling of information flow from vessels to neurons (i.e., VNC) and a potential contributor to the cognitive decline reported in BCAS mice. The observation that reduced A1R to A2_B_R mRNA ratios recovered by day 28 post-surgery in the somatosensory cortex but not in the hippocampus highlights the selective vulnerability of distinct brain regions to ischemic insults. Future studies addressing the dynamic changes in A1, A2_A_R, and A2_B_R subtypes in specific cell types will elucidate adenosine’s role in the NVU functional outcomes.

## Supplementary information

Supplementary Figure 1 **Basic membrane properties and input-output functions of cortical pyramidal neurons. A.** Resting membrane potential, **B.** input resistance, **C.** capacitance, and **D.** AP frequency (Hz) in sham (*n* = 57), BCAS 14d (*n* = 46) and BCAS 28d (*n* = 55) mice. (PDF 449 kb)
