# Supplementary figures and images for: Decreased parenchymal arteriolar tone uncouples vessel-to-neuronal communication in a mouse model of vascular cognitive impairment
Source: GeroScience. 2021 Jan 7;43(3):1405–22. doi: 10.1007/s11357-020-00305-x (PMC8190257; doi:10.1007/s11357-020-00305-x)

**A**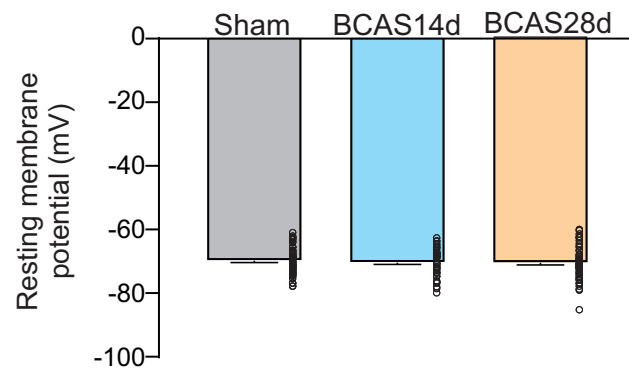**B**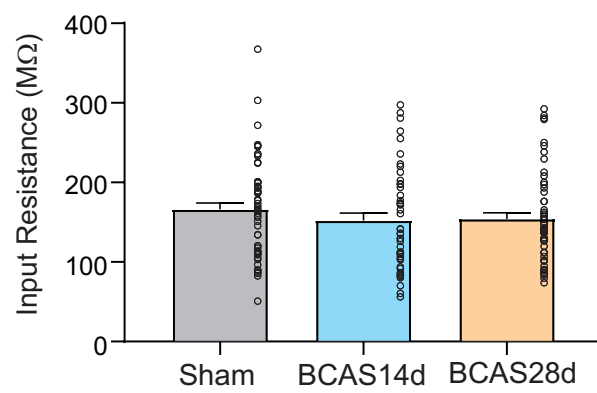**C**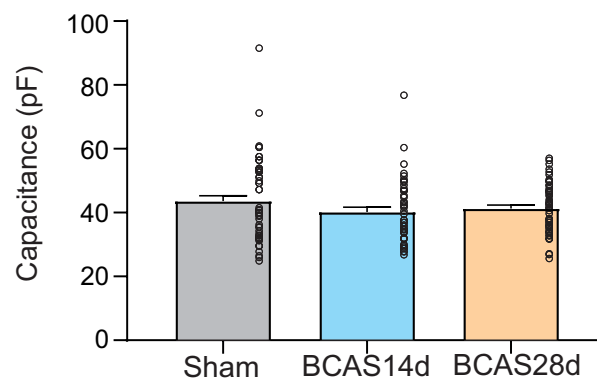**D**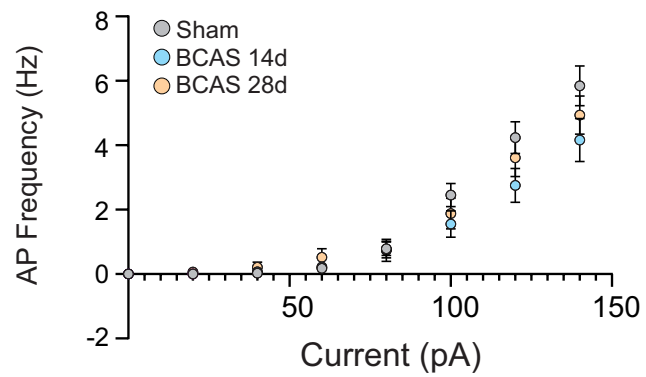

Supplement: Supplementary file 1 — Basic membrane properties and input-output functions of cortical pyramidal neurons. A. Resting membrane potential, B. input resistance, C. capacitance, and D. AP frequency (Hz) in sham (n = 57), BCAS 14d (n = 46) and BCAS 28d (n = 55) mice. (PDF 449 kb) [file 11357_2020_305_MOESM1_ESM.pdf]
